# Increasing rectum–prostate distance using a hydrogel spacer to reduce radiation exposure during proton beam therapy for prostate cancer

**DOI:** 10.1038/s41598-023-45557-7

**Published:** 2023-10-26

**Authors:** Tsukasa Narukawa, Norihiro Aibe, Masashi Tsujimoto, Takumi Shiraishi, Takuya Kimoto, Gen Suzuki, Takashi Ueda, Atsuko Fujihara, Hideya Yamazaki, Osamu Ukimura

**Affiliations:** 1 Department of Urology, Kyoto Prefectural University of Medicine, Kawaramachi-Hirokoji 465, Kyoto, Kyoto 602-8566 Japan; 2 Department of Radiology, Kyoto Prefectural University of Medicine, Kawaramachi-Hirokoji 465, Kyoto, Kyoto 602-8566 Japan

**Keywords:** Medical research, Urology

## Abstract

SpaceOAR, a polyethylene-glycol hydrogel, reduces rectal radiation exposure during radiation therapy for prostate cancer. Previously, our group reported the modified technique of hydrogel insertion, which achieves greater separated distance at prostate-apex. This study aimed to investigate the impact of separated distance at prostate-apex and our modifier technique, on radiation exposure reduction during proton beam therapy (PBT). We included 330 patients undergoing PBT with the relative biological effectiveness (RBE) of 63 Gray (Gy) for localized prostate cancer, and categorized them into groups 0 (no spacer, *n* = 141), 1 (separated distance of spacer at the prostate-apex level < 7.5 mm, *n* = 81), and 2 (distance ≥ 7.5 mm, *n* = 108). The rectal volumes to receive 30–60 Gy (RBE), was estimated and described as Rectal V30–60 (ml) in 10 Gy increments. The Rectal V30–60 (ml) was significantly lower in group 2 than in group 1, and in group 1 than in group 0. After propensity score matching, the multivariate logistic regression analysis revealed that the most significant factor to reduce radiation exposure was our modified technique of hydrogel insertion. Therefore, using a hydrogel spacer to expand the prostate–rectum distance not only at prostate-mid to prostate-base level but also at the prostate-apex level can reduce the radiation exposure in PBT for prostate cancer.

## Introduction

Prostate cancer is one of the most frequent malignancies in men worldwide, with 1,414,000 cases and 374,000 estimated deaths in 2020^1^. External beam therapy and brachytherapy are among the standard treatments for localized prostate cancer^2–5^; however, the adverse events (AEs) of rectal radiation exposure resulting from prostate–rectum proximity are significant clinical issues^6,7^. To reduce the issue of prostate–rectum proximity, researchers have reported several biomaterials used for increasing the distance between the prostate and the rectum to minimize rectal radiation exposure^8–10^. Polyethylene-glycol (PEG) hydrogel spacer (SpaceOAR; Boston Scientific Japan, Tokyo, Japan) is one of the materials approved by the US Food and Drug Administration and is designed to reduce rectal radiation exposure during radiation therapy for prostate cancer^11–15^. Creating at least 7.5 mm space between the prostate and the rectum at the prostate-mid level using SpaceOAR was reported to be a technical success in 96.6% of cases before the introduction of intensity-modulated radiation therapy (IMRT; 78 Gy delivered over an 8-week period)^16^. However, in clinical practice, the dilated distance between the prostate and the rectum at the prostate-apex level tends to be lower than that of prostate-mid and prostate-base levels. Given that the toxicity by radiation exposure generally depends on the proximity between the prostate and the rectum, the dilated distance at the prostate-apex level potentially exerts influence on the severity of rectal radiation exposure. We recently reported the feasibility and safety of a modified technique of inserting a spacer in achieving a greater prostate–rectum distance at the prostate-apex level^17^. However, we found few reports about the influence of expanding the distance between the prostate and the rectum using a hydrogel spacer on toxicity by radiation exposure^18^. Hence, this study aimed to investigate whether increasing the distance between the prostate and the rectum at prostate-apex level using a hydrogel spacer (SpaceOAR) reduces the rectal radiation exposure during PBT for prostate cancer. In addition, previous reports defined at least 7.5 mm space at the prostate-mid level was defined as technical success^16^, therefore, at least 7.5 mm space at prostate-apex was used as threshold in this study.

## Materials and methods

The Institutional Review Board (IRB) of Kyoto Prefectural University of Medicine approved this retrospective, single-center study, which conforms to the provisions of the Declaration of Helsinki (IRB number: ERB-C-1637). The inclusion criteria for the use of the hydrogel spacer were localized prostate cancers without suspicious of contact with the rectum. We enrolled 330 patients with localized prostate cancer who underwent PBT of 63 Gy (relative biological effectiveness RBE) in 21 fractions and postoperative MRI of the prostate before PBT introduction. The indication for treatment was judged during the PBT conference, which consists of department of urology and radiation therapy members, between May 2019 and February 2021. The spacer insertion was performed between August 2019 and August 2021. Androgen deprivation therapy was started before PBT, and it has been continued for certain periods according to the clinical stage or risk classification. All the patients, with written informed consent, underwent placement of gold fiducial markers to increase the accuracy for PBT targeting, and insertion of a hydrogel spacer (SpaceOAR) to reduce rectal radiation exposure was performed for eligible patients at the same time. These patients were categorized into three groups: group 0 (no spacer, *n* = 141), group 1 (rectum–prostate distance at the prostate-apex level of < 7.5 mm, *n* = 81), and group 2 (distance ≥ 7.5 mm, *n* = 108). Using preplanning computed tomography examination, we measured the rectal volumes to receive 30–60 Gy (RBE), described as Rectal V30–60 (ml) in 10 Gy increments. The hydrogel spacer remained in place for 3 months, and then hydrolyzing into liquid and absorbed in the body over several months. Hence, AEs (e.g., genitourinary gastrointestinal AEs and others such as cystitis, urethritis, dermatitis, hot flush, fatigue, and macrohematuria) appearing within 4 months after spacer implantation were evaluated and describes as acute AEs. The AEs appeared after that were also evaluated and, described as late AEs. The late AEs also included genitourinary, gastrointestinal AEs and others such as hot flash, sexual dysfunction (e.g., erectile dysfunction, ejaculation disorder, and decrease in semen), muscle weakness, fatigue, weight loss, joint pain, angina pectoris symptom, and pelvic discomfort.

The AEs of each patient was evaluated by the each attending radiologist who didn’t know the operator of spacer insertion, and the data was obtained from medical record database retrospectively. The AEs were categorized according to the National Cancer Institute Common Terminology Criteria for Adverse Events version 4.0.

### Delivery method of PBT

PBT was delivered using the real-time-image gated spot-scanning system with gold fiducial markers implanted in prostate^19^. The clinical target volume (CTV) included 3 mm margin around prostate and seminal vesicles. The CTV was modified based on anatomical boundaries or physician’s discretion and the volume of targeted seminal vesicles was dependent on the risk classification. For the robustness of plan, 3-mm lateral margin was used, and the proximal and distal margin of 3.5% range + 1 mm was used to reduce the range uncertainty^20^. Treatment plan was built as the 98% of CTV was encompassed by the 100% prescribed dose, or 63 Gy (RBE). Patients were instructed to empty their rectum as much as possible and to urinate 1 h before treatment. Daily patient alignments were achieved by first performing bone registration and then matching fiducial markers. Our proton delivery system only deliveres when the fiducial marker is within ± 2 mm of the planned position.

### Spacer insertion technique

The patients were placed in the lithotomy position. Using transrectal ultrasound scan (TRUS) guidance under local anesthesia with transperineal injection, we inserted the gold markers into the prostate. In inserting the spacer into the Denonvilliers space (fatty tissue between prostate and anterior rectal wall), the space was first expanded through saline solution injection. After confirmation of the proper location of the applicator needle for the spacer, 10 ml of PEG precursors were injected through the needle tip. In the conventional technique, the needle tip remained at the prostate-mid level during the injection, and all the procedures were performed under the real-time TRUS monitoring^16^. All PEG precursors must be injected within 10 s to form enough distance between the prostate and the rectum. However, in this conventional technique, the spacer would likely provide enough expansion in the prostate-mid and -base levels (where the needle tip is directed toward) but unlikely in the prostate-apex level.

However, in our reported modified technique, immediately after the hydrogel was confirmed to be injected in the distal end, the needle tip would be quickly moved back under the prostate-apex level, and hydrogel injection would be continued, resulting in enough hydrogel thickness throughout the Denonvilleier space under the prostate (Supplementary Fig. 1)^17^. If the needle tip was missed during the procedure, movement should be stopped at that time. The modified technique was not deviated from the manufacture’s instruction and performed within the scope of daily medical treatment. Within the study period, five different urologists (TN, TS, AU, KT, and MK) performed spacer injection. TN developed the novel modified technique after 18 cases of conventional method and only has applied since April 2020; other urologists performed the operation on only 5–26 cases and used the conventional method. The number of cases performed by each urologist and its technique were shown in supplementary Table 1. All the urologists were specialists of urology with enough experience and a license. None of the patients had hydrogel injection–related AEs of grade 3 or higher, but one in each group experienced grade 2 AEs (e.g., transient dysuria), which improved spontaneously within a few days.

### Measurement of the distance between the prostate and the rectum by an injected spacer

The distance between the prostate and the rectum expanded by the injected spacer was evaluated using the T2-weighted image of postoperative MRI obtained before PBT introduction, and the hydrogel was recognized as a hyperintense signal. In particular, the distance at the prostate-apex level was measured using the axial T2-weighted image of the MRI^21^, whereas that at the prostate-mid and prostate-base levels was measured using the midsagittal T2-weighted image slice of the MRI (Supplementary Fig. 2). The rectal wall infiltration of spacer (RWI) was also evaluated by using the axial T2-weighted image of MRI.

### Statistical analysis

We used chi-squared test for the qualitative data, and Mann–Whitney *U* test for the quantitative data. Simple Linear Regression was employed for the correlation analysis between separated distance at apex-prostate and Rectal V30–60 (mL). Propensity score matching was used to adjust and match the prostate volume between groups 1 and 2. In the matched groups, factors that aid in achieving at least 7.5 mm separated distance between the prostate and the rectum at the prostate-apex level were evaluated by univariate and multivariate logistic regression analyses. These factors included obesity (body mass index, < 25 or ≥ 25 kg/m^2^), insertion technique (modified or conventional), cT stage (≤ cT2 or ≥ cT3a), Gleason score (≤ 7 or ≥ 8), and prostate volume (< 50 or ≥ 50 ml).

Statistical data except for simple linear regression were analyzed using the statistical software EZR, which is based on the open-source R statistical software version 3.0.2^22^. GraphPad Prism8 software (Dotmatics, San Diego, CA, USA) was used for simple linear regression analysis. A *p* value of ≤ 0.05 was considered statistically significant, and all data are presented as median (interquartile range IQR).

### Ethical approval

The Institutional Review Board (IRB) of Kyoto Prefectural University of Medicine approved this retrospective, single-center study, which conforms to the provisions of the Declaration of Helsinki (IRB number: ERB-C-1637).

### Informed consent

All the patients provided written informed consent.

## Results

### Impact of hydrogel thickness at the prostate-apex level on the estimated rectal radiation exposure dose

**Figure 1:**
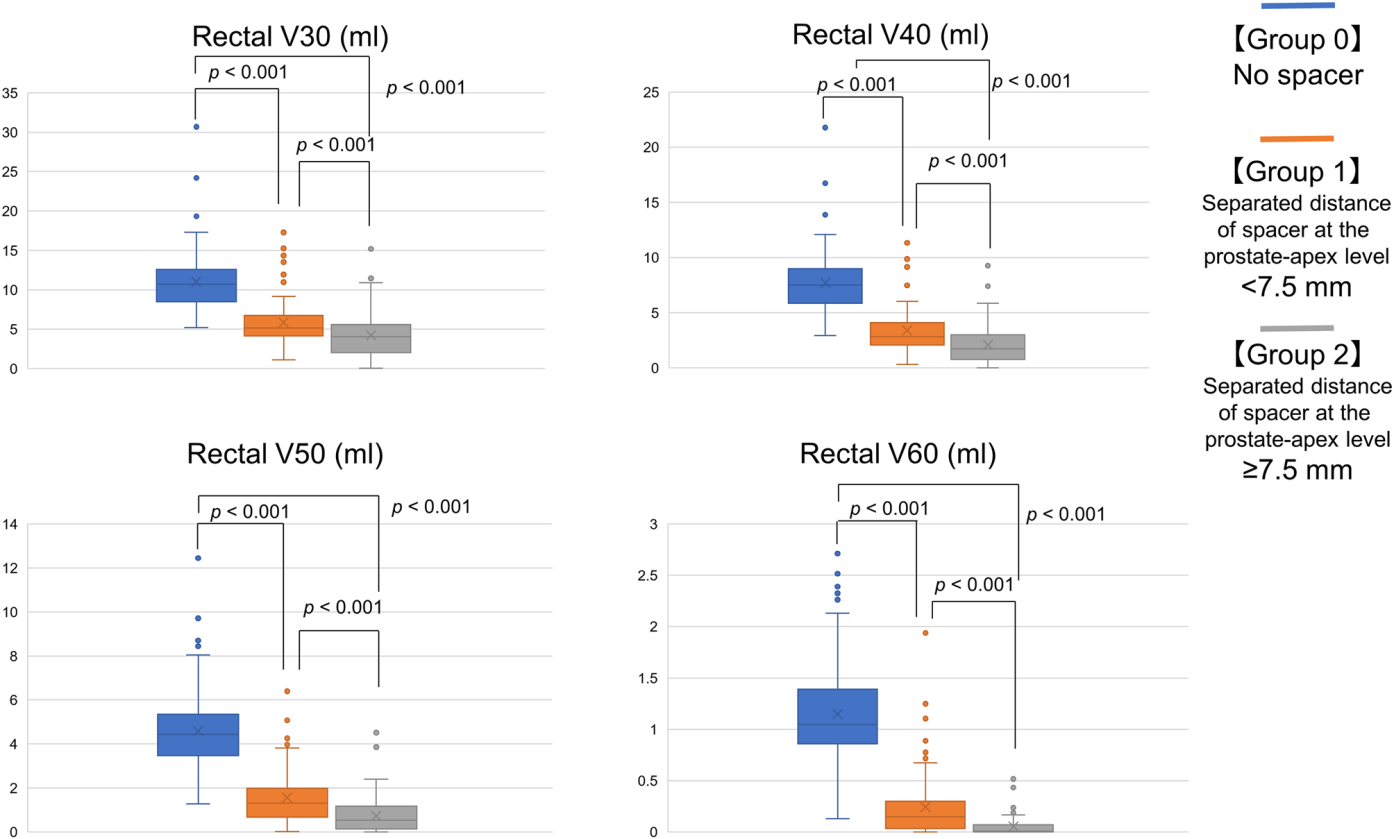
Median distribution of Rectal V30–60 (RBE) (cc) according to the separated distance at the prostate-apex level. The rectal radiation dose was lower in group 2 than in group 1, and in group 1 than in group 0, regarding Rectal V30–60 (RBE) (cc) (each: *p* < 0.001). RBE, relative biological effectiveness.

Figure 1 shows the median distribution of “Rectal V30–60 (ml)” among the three groups. The median distribution was lower in group 2 than in group 1 (*p* < 0.001), and in group 1 than in group 0 (*p* < 0.001), respectively.

### Correlation between the separated distance at prostate-apex and rectum radiation dose.

**Figure 2:**
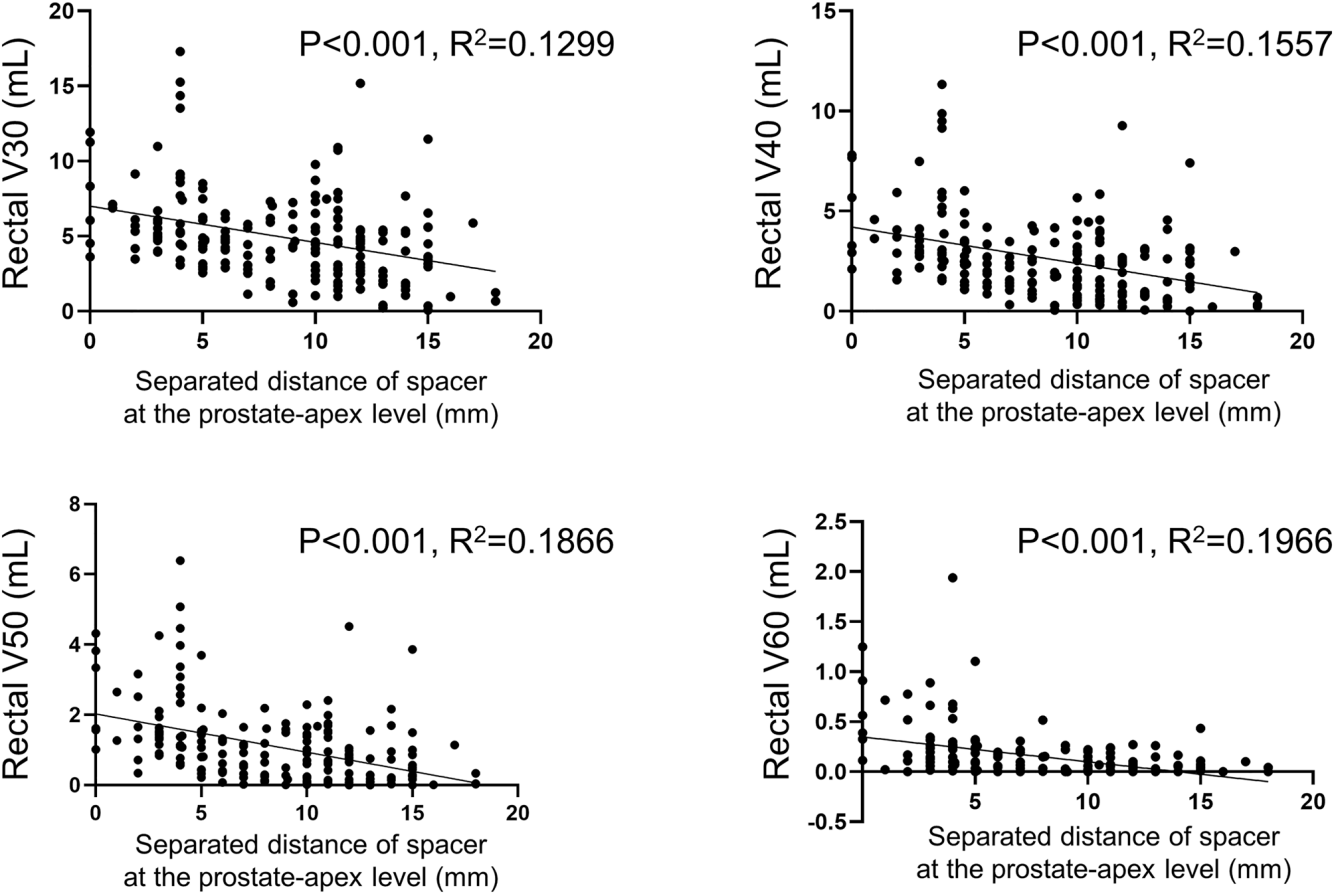
The regression distribution of the separated distance at prostate-apex (mm) and Rectal V30–60 (mL) in each spacer patient. The separated distance at prostate-apex was negatively correlated with the Rectal V30–60 (mL) (p < 0.001), respectively, in simple linear regression.

Figure 2 shows the regression distribution of the separated distance at prostate-apex and Rectal V30–60 cc in each spacer patient. The separated distance at prostate-apex was negatively correlated with the Rectal V30–60 (mL) (p < 0.001), respectively, in simple linear regression.

### Clinical characteristics and outcomes according to the separated distance of the spacer at the prostate-apex level

Table 1 summarizes the clinical characteristics and outcomes. None of the patients had acute AEs of grade 3 or higher. In this study period, one patient in group 0 developed the femoral neck fracture, one patient in group1 developed bladder cancer, and one patient in group 2 developed a stroke. In addition, one patient in group 1 and 2 died from hepatocellular carcinoma and lung cancer 27 and 30 months after spacer implantation, and no other G3 ≤ AEs occurred. Compared with group 1, group 2 had lower prostate volume (*p* = 0.005), lower rate of medication for urinary symptoms before PBT (p < 0.001), lower acute G2 genitourinary AE (*p* = 0.004) and late G1 gastrointestinal AE incidence (p = 0.004), and a higher rate of modified hydrogel insertion technique (*p* = 0.018), higher RWI incidence (p = 0.02), and greater hydrogel thickness at the prostate-mid and prostate-base levels (*p* < 0.001).

**Table 1:** Comparisons according to the created distance between the prostate and the rectum at the prostate-apex level.

Variable	No spacer	Spacer cases Distance expanded by a hydrogel spacer at the prostate-apex level		*p* value (Group 1 vs. Group 2
	Group 0 Reference	Group 1 < 7.5 mm	Group 2 ≥ 7.5 mm	
	*N* = 141	*n* = 81	*n* = 108	
Age (years)	73 (70–78)	72 (69.5–76)	72 (70–77.8)	0.53
BMI (kg/m^2^)	24.1 (22.4–25.9)	23.9 (22.4–25.9)	23.8 (22.4–25.9)	0.87
Diabetes, n (%)	20 (14.2%)	11 (13.6%)	10 (9.3%)	0.36
cT stage				
≤ cT2c, n (%)	4 (2.8%)	61 (75.3%)	77 (71.3%)	0.62
≥ cT3a, n (%)	137 (97.2%)	20 (24.7%)	31 (28.7%)	
Gleason score				
≤ 7, n (%)	53 (37.6%)	52 (64.2%)	58 (53.7%)	0.18
≥ 8, n (%)	88 (62.4%)	29 (35.8%)	50 (46.3%)	
Initial PSA (ng/ml)	12 (6.8–23.1)	9.7 (5.7–13.7)	8.7 (5.7–13.8)	0.81
Prostate volume (ml)	27 (20–38.5)	34 (23–45)	28 (18–38)	0.005*
Base line urinary status before PBT				
Medication for urinary symptoms, n (%)	36 (26%)	36 (44%)	19 (18%)	< 0.001
IPSS		9 (4–19)	6 (3.75–11)	0.10
Spacer insertion technique				
Modified, n (%)	–	34 (42%)	65 (60%)	0.018*
Conventional, n (%)	–	47 (58%)	43 (40%)	
Separated distance between the prostate and the rectum in each level				
At prostate-apex level	–	4 (3–5.6)	11 (10–13.8)	–
At prostate-mid level	–	8.8 (6.4–11.3)	12.7 (11.5–14)	< 0.001*
At prostate-base level	–	11.5 (8.8–12.7)	13.5 (11.5–15)	< 0.001*
Acute AEs				
Genitourinary AEs,				
G1, n (%)	75 (53%)	47 (58%)	66 (61%)	0.78
G2 ≤ , n (%)	57 (41%)	42 (52%)	33 (31%)	0.004*
Gastrointestinal AEs,				
G1, n (%)	6 (4.3%)	1 (1.2%)	2 (1.9%)	1
G2 ≤ , n (%)	1 (0.7%)	2 (2.5%)	2 (1.9%)	1
Others				
G1, n (%)	1 (0.7%)	6 (7.4%)	7 (6.5%)	1
G2 ≤ , n (%)	1 (0.7%)	1 (1.2%)	2 (1.9%)	1
RWI, n (%)	–	5 (6.2%)	20 (19%)	0.02*
Observation duration, (months)	32 (24–37)	31 (26–38)	32 (26–38)	0.82
Late AEs				
Genitourinary AEs				
G1, n (%)	75 (53%)	38 (47%)	55 (51%)	0.69
G2 ≤ , n (%)	41 (29%)	20 (25%)	23 (21%)	0.71
Gastrointestinal AEs				
G1, n (%)	29 (29%)	11 (14%)	2 (1.9%)	0.004*
G2 ≤ , n (%)	10 (7%)	1 (1.2%)	2 (1.9%)	1
Others				
G1, n (%)	20 (14%)	5 (6.2%)	11 (10%)	0.47
G2 ≤ , n (%)	5 (3.5%)	3 (3.7%)	3 (2.8%)	0.72

Results are presented as the median (IQR).

AE, adverse events; BMI, body mass index; IPSS, international prostate symptoms score; PBT, proton beam therapy; RWI, rectal wall spacer infiltration.

*Significantly different between groups 1 and 2 (*p* = 0.05).

After matched pair extraction on prostate volume, 138 patients were analyzed; their clinical characteristics and outcomes are enumerated in Table 2. The matched pair analysis also revealed that group 2 had a higher rate of modified hydrogel insertion technique (*p* < 0.001), lower rate of medication for urinary symptoms before PBT (p = 0.027), and a lower incidence of acute G2 genitourinary AEs (*p* = 0.03) and late G1 gastrointestinal AEs (p = 0.02) than group 1. Group 2 also had larger hydrogel thickness at the prostate-mid and prostate-base levels (*p* < 0.001) and smaller volumes in Rectal V30cc (*p* = 0.008) and V40–60 cc (*p* < 0.001) than group 1.

**Table 2:** Characteristics of matched patients in terms of prostate volume.

Variable	Distance expanded by a hydrogel spacer at the prostate-apex level		*p* value
	Group 1 < 7.5 mm	Group 2 ≥ 7.5 mm	
	*n* = 69	*n* = 69	
Age (years)	72 (70–76)	72 (69–76)	0.98
BMI (kg/m^2^)	23.9 (22.5–25.9)	24 (22.4–26)	0.87
Diabetes, n (%)	11 (15.9%)	8 (11.6%)	0.62
cT stage			
≤ cT2c	52	46	0.35
≥ cT3a	17	23	
Gleason score			
≤ 7	43	37	0.39
≥ 8	26	32	
Initial PSA (ng/ml)	9.70 (5.69–13.86)	9.87 (5.6–13.8)	0.97
Prostate volume (ml)	30 (23–38)	30 (22–39)	0.97
Base line urinary status before PBT			
Medication for urinary symptoms, n (%)	28 (41%)	15 (22%)	0.027*
IPSS	9 (4–14)	7 (4–11)	0.26
Spacer insertion technique			
Modified	29	56	< 0.001*
Conventional	40	13	
Separated distance between the prostate and the rectum in each level			
At prostate-apex level	4 (3–5.1)	11 (10–14)	-
At prostate-mid level	8.8 (6.4–11.3)	12.6 (11.5–14)	< 0.001*
At prostate-base level	11.9 (9.1–12.9)	12.9 (11.9–15.1)	< 0.001*
RWI, n (%)	4 (5.8%)	11 (16%)	0.10
Acute AEs			
Genitourinary AEs			
G1, n (%)	41 (59%)	36 (52%)	0.49
G2 ≤ , n (%)	38 (55%)	24 (35%)	0.03*
Gastrointestinal AEs			
G1, n (%)	1 (1.4%)	1 (1.4%)	1
G2 ≤ , n (%)	2 (2.9%)	2 (2.9%)	1
Others			
G1, n (%)	5 (7.2%)	5 (7.2%)	1
G2 ≤ , n (%)	1 (1.4%)	1 (1.4%)	1
Rectal volumes to receive 30–60 Gy (RBE) of proton beam therapy			
Rectal V30 (ml)	5.16 (4.17–6.25)	4.46 (2.85–5.87)	0.008*
Rectal V40 (ml)	2.83 (2.02–3.79)	2.33 (1.03–3.0)	< 0.001*
Rectal V50 (ml)	1.31 (0.63–1.65)	0.66 (0.21–1.24)	< 0.001*
Rectal V60 (ml)	0.146 (0.022–0.27)	0.015 (0–0.074)	< 0.001*
Observation duration (months)	32 (27–38)	31 (26–35)	0.58
Late AEs			
Genitourinary AEs			
G1, n (%)	32 (46%)	35 (51%)	0.73
G2 ≤ , n (%)	15 (22%)	17 (25%)	0.84
Gastrointestinal AEs			
G1, n (%)	9 (13%)	1 (1.4%)	0.02
G2 ≤ , n (%)	1 (1.4%)	1 (1.4%)	1
Others			
G1, n (%)	5 (7.2%)	8 (12%)	0.56
G2 ≤ , n (%)	2 (2.9%)	2 (2.9%)	1

Results are presented as median (IQR).

AE, adverse events; BMI, body mass index; IPSS, international prostate symptoms score; PBT, proton beam therapy; RBE, relative biological effectiveness; RWI, rectal wall spacer infiltration.

*Significantly different between groups 1 and 2 (*p* = 0.05).

### Logistic regression analysis of clinical factors for achieving at least 7.5 mm hydrogel thickness at the prostate-apex level

The univariate and multivariate logistic regression analyses among the 136 patients identified that the modified hydrogel insertion technique was the only factor that contributed to achieving at least 7.5 mm separated distance at the prostate-apex level (*p* < 0.001) (Table 3).

**Table 3:** Univariate and multivariate analyses of the *cumulative incidence* of achieving at least 7.5 mm separated distance at the prostate-apex level.

Variables	Case number	Success events	*U*nivariate analysis			Multivariate analysis		
			OR	95% CI	p value	OR	95% CI	p value
ALL	138	69						
Obesity								
BMI < 25 kg/m^2^	83	42	1	–	–	–	–	–
BMI ≥ 25 kg/m^2^	55	27	0.941	0.476–1.86	0.86	0.958	0.451–2.04	0.91
Methods								
Modified	85	56	1	–	–	–	–	–
Conventional	53	13	0.168	0.079–0.363	< 0.001	0.164	0.075–0.36	< 0.001*
cT stage								
≤ cT2	98	46	1	–	–	–	–	–
≥ cT3a	40	23	1.53	0.728–3.21	0.26	1.26	0.55–2.89	0.59
Gleason score								
≤ 7	80	37	1	–	–	–	–	–
≥ 8	58	32	1.43	0.725–2.82	0.301	1.57	0.734–3.34	0.25
Prostate volume (ml)								
< 50	124	62	1	–	–	–	–	–
≥ 50	14	7	1	0.331–3.02	1	0.764	0.224–2.61	0.67

BMI, body mass index; CI, confidence interval; OR, odds ratio.

## Discussion

Using logistic regression analysis, this study investigated the significant factors that aid in reducing radiation exposure on the rectal wall. Results showed that the modified hydrogel insertion technique was the only factor that had an impact on potential reducing the radiation exposure.

Danny Y et al. reported that injection of hydrogel spacer between the prostate and the rectum resulted in dose reductions to the rectum for > 90% of patients undergoing external beam radiation therapy for prostate cancer; interestingly, the median hydrogel thickness at the prostate-apex level (7.1 mm) was smaller than that at the prostate-mid level (9.4 mm)^21^. In fact, Fukumitsu et al. reported greater hydrogel thickness at the prostate-apex level, causing a smaller radiation exposure to the rectum^18^. Our group reported the feasibility and safety of the modified hydrogel insertion technique, which achieved greater hydrogel thickness at the prostate-apex level than the conventional technique^17^. We found that the estimated rectal radiation exposure dose was reduced according to the hydrogel thickness at the prostate-apex level. Taken together, considering that conventional methods tend to cause lesser hydrogel thickness at the prostate-apex level, increasing the hydrogel thickness at such level may reduce the rectal radiation exposure.

After the matched pair analysis between groups 1 and 2, our insertion technique was the only statistically significant factor that created an impact on achieving at least 7.5 mm hydrogel thickness. According to previous reports, procedure-associated AEs of grade 2 or more occurred in 3.3% of cases^23^, and among the recently reported AEs were severe rectal injury and spacer migration into the periprostatic venous plexus^24,25^. Therefore, evaluating the safety of the insertion technique is important. Given that TRUS was reported to be useful for the surgical navigation and diagnosis of prostate cancer^26^, monitoring the needle tip during injection by real-time TRUS is important to prevent complications related to the location of injection. Although our institute didn’t routinely evaluate spacer thromboembolism, there was no clinically significant thromboembolism, in this study period.

Despite the real-time TRUS guidance, modified technique might increase the rate of RWI due to the moving technique. In practice, the rate of RWI was higher in group 2, than group 1. In a previous report, the RWI incidence occurred in 6% of spacer cases, however, the incidence was not correlated with procedure-related adverse events or acute/late rectal toxicity^27^. In this study, all the patients completed the PBT treatment without any G3 ≤ AEs, with or without the RWI, hence, the influence of RWI was considered to be limited, in the real world.

Although hydrogel thickness at the prostate-apex level could lead to urinary dysfunction resulting from obstruction of the urethra close to the prostate-apex, the modified technique group only had one patient (1%) reported to have experienced transient dysuria. Nonetheless, the symptoms recovered within a few days. Considering that the rate of acute G2 genitourinary AEs was smaller in group 2 than in group 1, the acute genitourinary AEs might depend on not hydrogel thickness but the patients’ characteristics such as prostate volume and past history of neurogenic bladder. That is because, about the baseline urinary status, the IPSS and rate of medication for urinary symptoms were also lower in group 2 than group 1.

Thermal-ablation technologies such as cryoablation and microwave coagulation are reportedly effective for controlling clinically significant cancer^28^. Such thermal-ablation therapies need a safety margin between the target lesion and anterior rectal wall to avoid rectal injury from thermal energy. Similar to this study, our group reported the importance of sufficient hydrogel thickness for cryoablation and microwave coagulation therapy in a cadaver model^29,30^, and our modified hydrogel insertion technique could potentially broaden the adaptation not only for radiation therapy.

This study has several limitations, such as a small sample size, the retrospective study design, and the short follow-up period. Moreover, as a significant limitation, hydrogel thickness might depend on not only insertion technique, but also the operator’s experience and learning curve^31^, because only one operator (TN) performed the modified technique^1^, which accounted over half of the cases in this study. Even if that were true, an ingenuity for achieving greater distance is important, because, a greater distance between the prostate and the rectum was associated with a greater reduction of the rectal radiation exposure dose, and which could potentially reduce the late gastrointestinal AEs. Interestingly, in spite of a short follow-up period, as a late gastrointestinal AE, G2 radiation proctitis only occurred in 6 patients of group 0, and G1 radiation proctitis rate was higher in group 1 than in group 2 (8.6% and 0.9%, respectively). Thus, the distance between the prostate and the rectum should be expanded to mitigate the rectal radiation dose during radiation therapy for prostate cancer, and which could potentially decrease the late gastrointestinal AEs. Recently, SpaceOAR Vue (Boston Scientific Japan, Tokyo, Japan) and Hyaluronic Acid Spacer are also used for radiation therapy of prostate cancer as a spacer. The modified technique is expected to be useful for their spacer insertion^32,33^. To our knowledge, this study is the first to investigate the impact of expanding the distance between the prostate-apex and the rectum using a hydrogel spacer for prostate cancer before PBT.

## Conclusions

An ingenuity of expanding the distance between the prostate and the rectum using a hydrogel spacer not only at the prostate-mid to prostate-base level but at the prostate-apex level can reduce the radiation exposure to the rectum during radiation therapy for prostate cancer.

### Supplementary Information

Supplementary Information.

## Data Availability

The authors confirm that the data supporting the findings of this study are available within the article.
