# Supplementary material for: Increasing rectum–prostate distance using a hydrogel spacer to reduce radiation exposure during proton beam therapy for prostate cancer
Source: Sci Rep. 2023 Oct 26;13:18319. doi: 10.1038/s41598-023-45557-7 (PMC10603046; doi:10.1038/s41598-023-45557-7)

**Increasing rectum–prostate distance using a hydrogel spacer to reduce radiation exposure during proton beam therapy for prostate cancer**

Tsukasa Narukawa*^,#,1^, Norihiro Aibe^#,2^, Masashi Tsujimoto^#,1^, Takumi Shiraishi^1^ , Takuya Kimoto^2^, Gen Suzuki^2^, Takashi Ueda^1^, Atsuko Fujihara^1^, Hideya Yamazaki^2^, and Osamu Ukimura^1^

**Supplementary Table 1**

| The number of cases and insertion technique performed by each urologist | | | | | |
| --- | --- | --- | --- | --- | --- |
| Phisician  Technique | TN | TS | AU | KT | MK |
| Conventional | 18 | 21 | 19 | 5 | 26 |
| Modified | 99 | - | - | - | - |

**Supplementary figure legends**

Supplementary Figure 1. Hydrogel spacer insertion technique. In the conventional technique, the intraoperative location of the injection needle tip was fixed to maintain at the prostate-mid level. In the new modified technique, the injection needle tip was continuously moved from the prostate-mid level back to the prostate-apex level under real-time ultrasound guidance, making the spacer thicker throughout the Denonvilliers space including under the prostate-apex.

Supplementary Figure 2. Measurement of the spacer thickness in postoperative MRI. Hydrogel was recognized as a hyperintense T2-weighted MRI signal. The separated distance at the prostate-apex level was measured using the axial image slice closest to the prostate-apex level. The mid-level was the halfway between the apex and the base, and the separated distance at the mid and base levels was measured using the midsagittal image slice of the MRI. MRI, magnetic resonance imaging; T2WI, T2-weighted image.

**Supplementary figure 1**


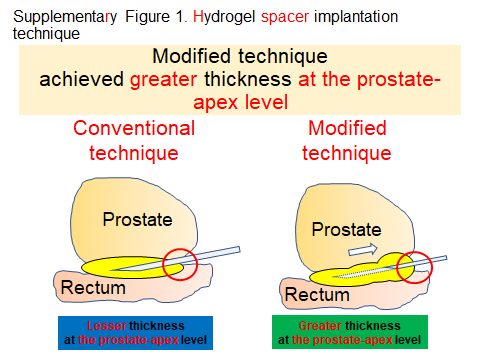


**Supplementary figure 2**


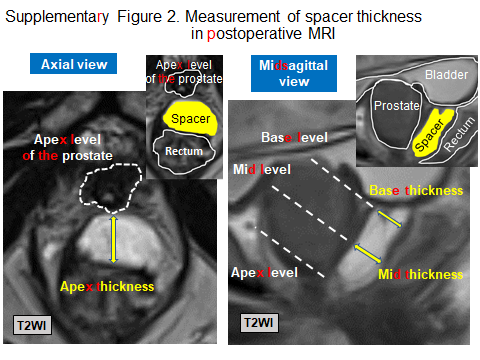

Supplement: Supplementary file 1 — Supplementary Information. [file 41598_2023_45557_MOESM1_ESM.docx]
